# SARS-Cov-2 small viral RNA suppresses gene expression via complementary binding to mRNA 3’ UTR

**DOI:** 10.17912/micropub.biology.000790

**Published:** 2024-01-18

**Authors:** Haley A Delcher, Jeffrey D DeMeis, Nicole Ghobar, Noel L Godang, Sierra L Knight, Shahem Y Alqudah, Kevin N Nguyen, Brianna C Watters, Glen M Borchert

**Affiliations:** 1 Department of Pharmacology, College of Medicine, University of South Alabama, Mobile, AL; 2 Department of Biology, College of Arts and Sciences, University of South Alabama, Mobile, AL

## Abstract

SARS-CoV-2 (SC2) has been intensely studied since its emergence. However, the mechanisms of host immune dysregulation triggered by SC2 remain poorly understood. That said, it is well established that many prominent viral families encode microRNAs (miRNAs) or related small viral RNAs (svRNAs) capable of regulating human genes involved in immune function. Importantly, recent reports have shown that SC2 encodes its own svRNAs. In this study, we have identified 12 svRNAs expressed during SC2 infection and show that one of these svRNAs can regulate target gene expression via complementary binding to mRNA 3’ untranslated regions (3’UTRs) much like human microRNAs.

**Figure 1. SARS-CoV-2 svRNA-29094 mimic suppresses target gene in PC3 cells:**
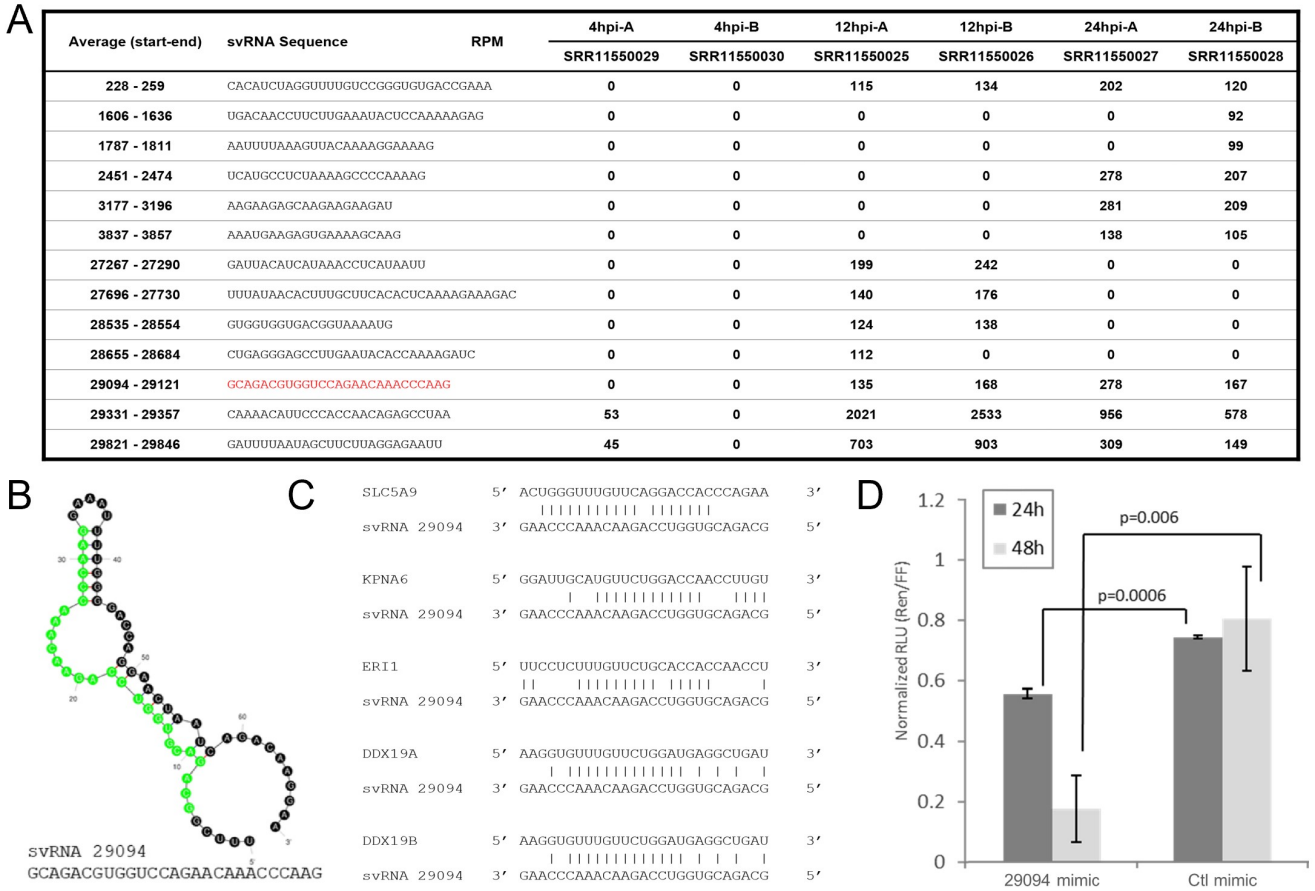
( **A** ) 12 svRNA sequences expressed in SARS-CoV-2 infected Calu-3 cells. Average start-end (position in the SC2 NC_045512 genome) alongside corresponding svRNA sequence (+ strand) are shown. Expression (in Reads Per Million) of individual svRNA sequences are indicated under the corresponding SRR Identifiers (Wyler et al. 2021). ( **B** ) mFold predicted secondary structure of svRNA-29094 hairpin (Zuker 2003). The excised svRNA is highlighted in green and included below the hairpin. ( **C** ) Alignment of putative target mRNA 3’UTRs with the svRNA-29094 sequence. ( **D** ) PC3 cell lysates were collected at 24- and 48-hours after co-transfection of a small RNA mimic and a psiCHECK-2 reporter expressing Renilla luciferase mRNA harboring the SLC5A9 target site (shown in C) in its 3’UTR. Graph depicts Relative Light Units (RLU) of Renilla luciferase normalized to firefly luciferase (internal control independently expressed from psiCHECK-2). 29094 mimic, commercially synthesized small RNA identical to the svRNA 29094 sequence depicted in B. Ctl mimic, standard Dharmacon negative control microRNA mimic. Error bars indicate standard deviation (n=3). P-values were obtained using a standard t-test.

## Description

Over the past 4 years, SARS-CoV-2 (SC2) has killed millions of people and has upended the global economy. SC2 belongs to the Coronaviridae family of viruses and has been classified as a positive sense, single-stranded RNA virus. Although asymptomatic in some patients, SC2 infection can lead to the development of coronavirus disease 19 (COVID-19), a life-threatening inflammatory illness that primarily affects the lungs (Zhang et al. 2020) . The severity of COVID-19 is largely reliant upon the ability of SC2 to replicate efficiently while simultaneously shutting down the host immune system (Chang et al. 2021) . Despite being intensely studied since its discovery, much is still unknown about the elements of the virus that enable it to dysregulate the host immune response in severe cases of COVID-19. That said, it is well established that many prominent viral families such as the Herpes and Papilloma virus families encode microRNAs (miRNAs) or related short viral RNAs (svRNAs) capable of regulating human genes involved in immune function (Samols et al. 2007; Sadri Nahand et al. 2020) . MiRNAs are small (~22nt) noncoding RNA that participate in post-transcriptional gene regulation by complementary binding to the 3’UTR of their respective target mRNAs with some miRNAs possessing the capacity to regulate dozens of target genes in a single controllable event (Lee et al. 1993) . Importantly, recent reports have now indicated that SC2 encodes its own svRNAs, and two other groups have already shown that the principle svRNA examined in this work, svRNA-29094, is specifically excised and highly expressed during SC2 infection (Meng et al. 2021; Neeb et al. 2022) .

Analysis of small RNA transcriptomes corresponding to Calu-3 cells at 1-, 12-, and 24-hours post-infection (hpi) with SC2 (Wyler et al. 2021) found ~95% of resultant small RNA sequences mapped to the human genome while < 0.4% aligned to the SC2 genome. Strikingly, we observed that roughly 92% of these viral small RNA reads mapped to one of 12 specific genomic positions, are ~20 nt in length and encoded on the positive strand and had peak expressions ranging between ~100 and 2,000 RPM ( **Figure 1A** ). We selected svRNA-29094 ( **Figure 1B** ) for further analysis primarily based on (1) its predicted target sequence alignments ( **Figure 1C** ) and (2) previous reports confirming its expression in SC2-infected cell lines and patients (Meng et al. 2021; Neeb et al. 2022) . To confirm the ability of svRNA-29094 to regulate its predicted targets, we performed co-transfections of small RNA mimics and a psiCHECK-2 reporter expressing Renilla luciferase mRNA harboring the SLC5A9 target site (shown in Figure 1C ) in its 3’UTR. Luciferase expression was measured at 24- and 48-hours post-transfection and ~27% and ~79% decreases (respectively) in Renilla activity were observed in cells treated with svRNA-29094 mimic as compared to cells transfected with mimic controls ( **Figure 1D** ).

Viruses such as SC2 remodel host gene expression through a variety of mechanisms including post-transcriptional gene regulation. SvRNAs have now been implicated in the regulation of a variety of different biological pathways related to infection and host immune evasion. Additionally, svRNAs have recently been identified to play important roles in viral propagation in SARS-CoV-1, influenza, and hepatitis infections (Meng et al. 2021) . For example, much like with SC2, SARS-CoV-1 infection frequently results in an exaggerated inflammatory response, significant lung pathology, and is associated with a high rate of mortality (Channappanavar and Perlman 2017) . The relevance of specific svRNAs in SARS-CoV-1 pathology was previously established, when ten ~20 nt svRNAs were discovered in deep sequenced RNAs from infected mice lungs. Importantly, antagomiR-based inhibition of one of these, svRNA-N, can significantly reduce pro-inflammatory cytokine expression and lung pathology in vivo (Morales et al. 2017) . These results demonstrate that (at least) this svRNA could directly contribute to SARS-CoV-1 pathogenesis, and also suggests that antagomiRs targeting other coronavirus svRNAs could potentially prove of value as a novel form of potent antiviral.

That said, multiple groups have now independently reported the expression of SC2 svRNAs, although their cellular functions remain almost entirely unresolved (Meng et al. 2021) . In this study, we have identified 12 small viral RNAs (svRNAs) expressed during SC2 infection and find at least one of these svRNAs can regulate target gene expression via complementary binding to mRNA 3’ untranslated regions. In short, our data confirms the ability of a svRNA-29094 mimic to suppress the expression of a mRNA bearing a 3’UTR target site, and as such, to our knowledge, this study is the first to confirm miRNA-like target mRNA repression by SC2 svRNAs. Furthermore, although we only directly confirmed the ability of svRNA-29094 to repress the intestinal sodium/glucose cotransporter SLC5A9 target site (Tazawa et al. 2005) , similarly strong alignments were identified with KPNA6 (known to facilitate nuclear translocation of interferon regulatory factor 3 and inhibited by SC2 membrane protein (Zhang et al. 2021) ), ERI1 (known to regulate aspects of Influenza A mRNA transcription (Declercq et al. 2020) ), DDX19A (known to detect and bind viral RNA to activate the NLRP3 inflammasome (Li et al. 2015) ), and DDX19B (a component of the nuclear pore complex known to interact with Influenza A viral polymerase (Munier et al. 2013) ) ( **Figure 1C** ) further supporting a role for svRNA-29094 in remodeling host gene expression related to infection and host immune evasion.

In conclusion, we suggest (1) the ability of svRNA-29094 to suppress the expression of a mRNA bearing a complementary 3’UTR target site, (2) the presence of putative svRNA-29094 target sites in several host genes related to infection and host immune evasion, and (3) the identification of svRNA-29094 expression during SC2 infection in both cell culture models and patient samples by three independent laboratories (Meng et al. 2021; Neeb et al. 2022) , strongly supports further characterization of svRNA-29094 and evaluation of its potential to serve as a novel therapeutic target.

## Methods

**Sequence analyses**

To identify svRNAs expressed during SC2 infection, we obtained next-generation small RNA deep sequencing libraries corresponding to Calu-3 cells infected with an SC2 patient isolate 1, 12, and 24 hours post-infection (Devadoss et al. 2021 May 18; Wyler et al. 2021; Devadoss et al. 2022) . Alignments between the SC2 genome and individual small RNA-seq reads were performed on the Alabama Supercomputer Center SGI UV 2000 and DMC cluster and obtained via Basic Local Alignment Search Tool (BLAST+) using the following parameters: 100% identity, word size = 6, un-gapped, and evalue = 0.001 (Camacho et al. 2009). All accepted BLAST+ alignments were restricted to perfect matches (100% identity) between 16 and 32 nts. The frequency of alignments to each position across the full-length genome was calculated by counting reads defined as ≥16 nts, perfect matches (100% identity) and svRNAs called as previously defined and employed (Roberts et al. 2013; Huang et al. 2014; Roberts et al. 2014; Amin et al. 2016; Huang, Eilbeck, et al. 2016; Cardin and Borchert 2017; Barnhill et al. 2019; Houserova et al. 2021; Coley et al. 2022; Godang et al. 2023) . Publicly available next-generation small RNA deep sequencing libraries were obtained from the NCBI Sequence Read Archive (SRA) (www.ncbi.nlm.nih.gov/sra/). These included SRR1155025, SRR1155026, SRR1155027, SRR1155028, SRR1155029, and SRR1155030 (Wyler et al. 2021) . Putative human 3’UTR targets were identified for svRNAs using an in-house BLAST-based pipeline previously defined and routinely employed by our lab (Borchert et al. 2009; Borchert et al. 2011; Filshtein et al. 2012; Huang, Gutierrez, et al. 2016; Chevalier and Borchert 2017; Patterson et al. 2017; Roberts and Borchert 2017; Chen et al. 2018; Roberts et al. 2018; Alexander B. Coley et al. 2022) .

**Luciferase assays**

Antisense reporters were constructed by oligonucleotide primer extension PCR in 50 µl reactions at standard concentrations (1.5 mM MgCl _2_ , 0.2 mM dNTP, 1x Biolase PCR buffer, 2.5 U Taq (Bioline USA, Inc., Randolph, MA), 0.1 µM each primer) and using standard cycling parameters (95°C - 2 min, (95°C - 40 sec, 58°C - 40 sec, 72°C - 40 sec) x 35 cycles, 72°C - 2 min) with primers containing 5’ Xho-I and 3’ Not-I restriction enzyme sites. Primer sequences were:

Forward: ACTCGAGAAGTCAGCACACAAAGCCAAAAACTGGGTTTGTTCAGGACCACCCAGAAGAAGAAGAGCAAGAAGAAGATTG

Reverse: TGCGGCCGCAACCAGCCTCATCCACGCACAATTTGGTGCCACTTCTGCTGCTCCAATCTTCTTCTTGCTCTTCTTCTTCTGGG

Following PCR amplification and subsequent digestion, amplicons were ligated into the Renilla luciferase 3’UTR of psiCHECK-2 (Promega) vector linearized with Xho-I and Not-I. The prostate cancer PC3 cell line was obtained from GenLantis (San Diego, CA) and cultured in a humidified atmosphere with 5% CO2 at 37°C in MEM (Mediatech, Herndon, VA) supplemented with 10% fetal bovine serum (Hyclone, Logan, UT), 25 mg/ml streptomycin and 25 I.U. penicillin (Mediatech). PC3 cells were seeded in a 96-well plate (10,000 cells per well) and allowed to adhere for 4 hours. Then cells were co-transfected with 500 ng of reporter plasmid and 10 pmol of svRNA mimic following lipofectamine 2000 (Invitrogen) protocol. Cells were lysed in the plate according to standard Dual-Glo Luciferase Reporter System (Promega, Madison, WI) at 24h and 48h post-transfection. Firefly and Renilla luciferase were measured sequentially at each time point using a 96-well plate luminometer (SpectraMax iD5 Multi-mode microplate reader (Molecular Devices) and normalized to an empty vector.
